# Methods and indicators for measuring patterns of human exposure to malaria vectors

**DOI:** 10.1186/s12936-020-03271-z

**Published:** 2020-07-13

**Authors:** April Monroe, Sarah Moore, Fredros Okumu, Samson Kiware, Neil F. Lobo, Hannah Koenker, Ellie Sherrard-Smith, John Gimnig, Gerry F. Killeen

**Affiliations:** 1 Johns Hopkins Center for Communication Programs, PMI VectorWorks Project, Baltimore, MD USA; 2 University of Basel, Basel, Switzerland; 3 Swiss Tropical and Public Health Institute, Basel, Switzerland; 4 Environmental Health and Ecological Sciences Department, Ifakara Health Institute, Ifakara, Tanzania; 5 School of Public Health, Faculty of Health Sciences, University of the Witwatersrand, Parktown, Republic of South Africa; 6 Institute of Biodiversity, Animal Health and Comparative Medicine, University of Glasgow, Glasgow, UK; 7 Eck Institute for Global Health, University of Notre Dame, Notre Dame, IN USA; 8 MRC Centre for Global Infectious Disease Analysis, Department of Infectious Disease Epidemiology, Imperial College London, Norfolk Place, London, W2 1PG UK; 9 Division of Parasitic Diseases, Centers for Disease Control and Prevention, Atlanta, GA USA; 10 Department of Vector Biology, Liverpool School of Tropical Medicine, Liverpool, UK; 11 School of Biological, Earth & Environmental Sciences and Environmental Research Institute, University College Cork, Cork, Republic of Ireland

**Keywords:** Insecticide-treated nets, Human-vector interaction, Human-vector contact, Exposure, Residual malaria transmission, Outdoor biting, Outdoor transmission

## Abstract

**Background:**

Effective targeting and evaluation of interventions that protect against adult malaria vectors requires an understanding of how gaps in personal protection arise. An improved understanding of human and mosquito behaviour, and how they overlap in time and space, is critical to estimating the impact of insecticide-treated nets (ITNs) and determining when and where supplemental personal protection tools are needed. Methods for weighting estimates of human exposure to biting *Anopheles* mosquitoes according to where people spend their time were first developed over half a century ago. However, crude indoor and outdoor biting rates are still commonly interpreted as indicative of human-vector contact patterns without any adjustment for human behaviour or the personal protection effects of ITNs.

**Main text:**

A small number of human behavioural variables capturing the distribution of human populations indoors and outdoors, whether they are awake or asleep, and if and when they use an ITN over the course of the night, can enable a more accurate representation of human biting exposure patterns. However, to date no clear guidance is available on what data should be collected, what indicators should be reported, or how they should be calculated. This article presents an integrated perspective on relevant indicators of human-vector interactions, the critical entomological and human behavioural data elements required to quantify human-vector interactions, and recommendations for collecting and analysing such data.

**Conclusions:**

If collected and used consistently, this information can contribute to an improved understanding of how malaria transmission persists in the context of current intervention tools, how exposure patterns may change as new vector control tools are introduced, and the potential impact and limitations of these tools. This article is intended to consolidate understanding around work on this topic to date and provide a consistent framework for building upon it. Additional work is needed to address remaining questions, including further development and validation of methods for entomological and human behavioural data collection and analysis.

## Background

Insecticide-treated nets (ITNs) have accounted for an estimated two-thirds of malaria cases prevented in the past decade [1]. However, their effectiveness is limited against mosquitoes that feed when people are outdoors, or indoors but awake and active. Furthermore, the scale-up of ITNs can contribute to shifts in species composition, as well as shifts in vector behaviour (e.g. toward early evening and early morning biting, increased outdoor resting and biting, and more frequent feeding upon animals) which may further attenuate vector control impact [2–4].

Quantifying and characterizing gaps in personal protection against mosquitoes, defined as the proportional reduction of biting exposure an individual experiences as a direct result of personal use of a protection measure, requires information on the behaviours of vectors and humans, as well as when and where they intersect. Methods for factoring spatiotemporal interactions between vectors and humans into biting exposure estimates were developed as early as the late 1960s and early 1970s [5–7]. A number of more recent articles have also underscored the importance of including human behaviour when investigating biting risk, and the value of biologically meaningful coverage indicators [8–10]. Despite the importance of human behaviour to understanding malaria transmission dynamics, relatively few studies have investigated it [11, 12].

A review of published literature between 2000 and 2017 identified fewer than a dozen studies in sub-Saharan Africa that integrated entomological and human behavioural data to enable more meaningful interpretation [11, 12]. Likewise, a systematic review and meta-analysis of mosquito feeding behaviour identified a surprising absence of data on human location and sleeping patterns needed to quantify risk of mosquito biting [12]. The review identified 250 data sets measuring mosquito biting time across Africa but only 22 of these data sets had documented human location, only seven documented human sleeping patterns, and only three had collected necessary human and vector data in the same time and place [12].

**Fig. 1:**
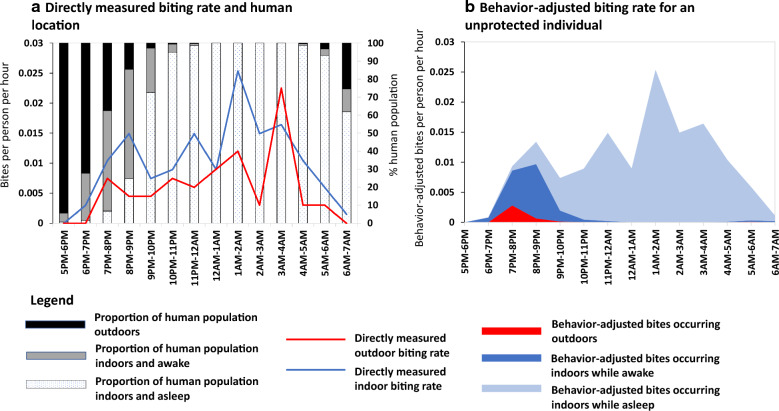
Example of directly measured and behaviour-adjusted estimates of human exposure to malaria vectors from Asembo, western Kenya in 2011. Mosquito biting data was collected using human landing catches from June through July 2011 and human behavioural data was collected using a cross-sectional survey conducted from July through August 2011 [52]. Series A shows the proportion of the human population (1) outdoors (2) indoors and awake, and (3) indoors and asleep throughout the night, overlaid with directly measured indoor and outdoor biting rates for *Anopheles arabiensis*. Based on biting density alone, the estimated percentage of vector bites occurring indoors = 63%. Series B integrates vector and human behaviour data to show behaviour-adjusted cumulative exposure to vector bites for an unprotected individual. The percentage of vector bites occurring indoors for an unprotected individual $\left( {\pi_{I,\,u}}\right)$ = 97% and the percentage occurring while asleep indoors for an unprotected individual $\left( {\pi_{S,u}}\right)$ = 84%

Crucially, calculation methods for weighting exposure estimates according to human location can alter interpretations when compared to those suggested by entomological observations alone [13–16]. For example, for a mosquito population that feeds both indoors and outdoors, the overwhelming majority of exposure events for an unprotected person may still occur indoors if mosquitoes actively seek blood throughout the night when most people are asleep inside their houses (Fig. 1).

Despite their utility, such quantitative indicators of when and where interactions between humans and vectors occur remain under-utilized, and no clear guidance is available on what data should be collected, what indicators should be reported, or how they should be calculated. Further, what little methodological literature exists is somewhat scattered, with inconsistent definitions and notation in different reports at different development stages of the methodology [11].

Beyond the implications for personal protection from mosquito bites, such numerical estimates for gaps in practically-achievable personal protection are also crucial determinants of how well ITNs function to control vector populations due to mosquito contact with the insecticides applied to them [3, 8, 17, 18]. The strong preferences for human blood that make some African *Anopheles* such efficient malaria vectors also render them vulnerable to population control with insecticidal personal protection measures like ITNs [2, 3, 8, 19]. Reduced vector population abundance, survival rate, feeding frequency and human-feeding probability, underpin equitable community-level *mass effects* that account for much of the benefits of widespread ITN use [2, 20, 21]. Successful suppression of a vector population depends directly upon the extent of personal protection ITNs provide for two reasons:

1. The degree of personal protection not only influences rates of human exposure to mosquitoes but also rates of mosquito exposure to the active ingredients of ITNs [22].
2. Personal protection is often what motivates end users of ITNs and drives utilization rates once access is provided [23].

However, vector population suppression is a much more complex phenomenon and is also limited by other vector behaviours such as feeding on animals [2, 24, 25], early exit from houses [26], and physiological resistance to insecticides [27, 28], none of which is within the scope of this article. Instead, this article focuses specifically on how best to measure and interpret behavioural determinants of personal protection in the field. Building on existing approaches in the literature, this article presents an integrated perspective on relevant indicators of human-vector interactions, the critical entomological and human behavioural data elements required to quantify human-vector interactions, and recommendations for collecting and analysing such data.

## Methods for measuring human-vector interaction

### Indicators of human-vector interaction patterns

Human-vector indicators can provide a clearer picture of where (indoors or outdoors) and when (time of night) exposure to malaria vectors occurs by accounting for human location and intervention use throughout the night. Relevant indicators include:

1. The proportion of vector bites occurring indoors for an unprotected individual $\left( {\pi_{I,u} } \right)$, which represents the maximum possible personal protection any indoor intervention could provide.
2. The proportion of vector bites occurring indoors during sleeping hours, for an unprotected individual $\left( {\pi_{S.u} } \right)$, which represents the maximum possible personal protection any intervention targeting sleeping spaces could provide.
3. The proportion of all vector bites prevented by using an ITN $\left( {P_{S}^{*} } \right)$, which represents the protection provided against vector bites to someone using an ITN during sleeping hours.
4. The proportion of remaining exposure occurring indoors for a protected user of an ITN $\left( {\pi_{I,p} } \right)$, which represents how much remaining (residual) exposure occurs indoors for an individual who uses an ITN. This indicator is particularly useful for understanding where malaria transmission is occurring once high coverage with ITNs is achieved and the relative merits of adding supplemental interventions that act indoors and/or outdoors in that context.
5. The population-wide mean personal protection against biting exposure provided by community-level coverage of humans with ITNs $\left( {P_{S,C}^{*} } \right)$, which represents the community average level of personal protection, accounting for the proportion of people who use an ITN each night while asleep. This summary indicator is particularly useful for adjusting field measurements of mosquito biting rates to account for this community-wide mean level of personal protection.

**Fig. 2:**
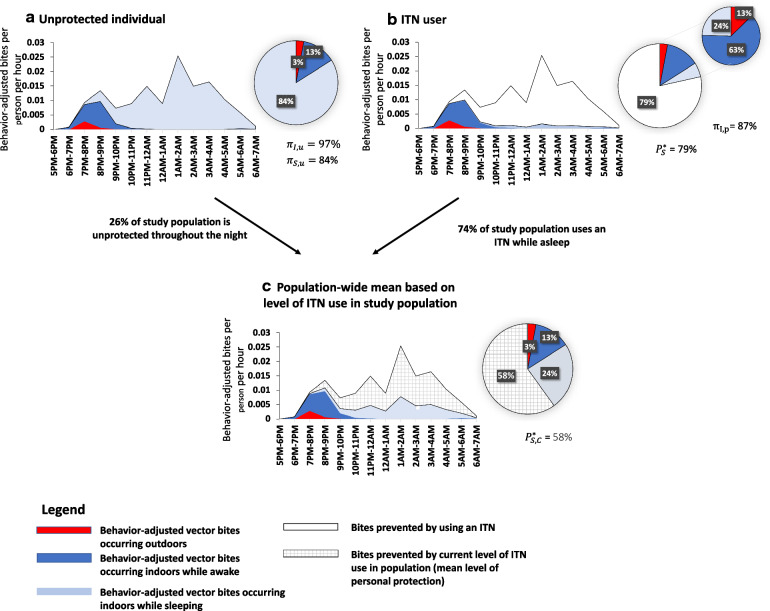
Example of indicators calculated using vector and human behaviour data from Asembo, western Kenya in 2011 [52]. Series A shows the behaviour-adjusted estimates of exposure to *Anopheles arabiensis* bites for an unprotected individual. Series B shows behaviour-adjusted estimates of exposure to vector bites for an ITN user. ITNs were assumed to prevent approximately 94% of bites while in use based on reference estimates from experimental hut trials. The percentage of all vector bites prevented by using an ITN $\left( {P_{S}^{*} } \right)$ = 79% and the proportion of remaining exposure occurring indoors for a protected user of an ITN $\left( {\pi_{I,p} } \right)$ = 87%. Series C shows the population-wide mean exposure to vector bites. In this site the proportion of the population that reported using an ITN while asleep the previous night was 74% (arrows). Therefore, the population-wide mean personal protection against biting exposure given the reported community-level coverage of people using an ITN $\left( {P_{S,C}^{*} } \right)$ is 58%. An Excel file demonstrating how these indicators were calculated is included as Additional file 1

While indicators 3–5 focus on the personal protection provided by ITNs, similar indicators may be calculated for mosquito-proofed housing, and other personal protection measures such as repellents or treated clothing. All indicators, when possible, should be disaggregated by vector species and by human population groups (e.g. by sex and age categories) as behaviour can vary across these species and groups. Equations for calculating population-level indicators of human-vector interaction are presented in Box 1 and an example using vector and human behaviour data from Asembo, western Kenya is used to comparatively illustrate these indicators in Fig. 2.

Box 1: Equations for calculating summary indicators of human-vector interaction patterns In order to maintain consistency and enable easy comparison with field-relevant literature, the following notation provides an updated, harmonized, and clarified version of that most commonly used in published articles quantifying human-vector interactions. However, examples of alternative notation for conceptually similar parameters can be found in the mathematical modeling literature and are also valid. In the notation presented here, “π” refers to the average proportion of human exposure to vector bites that occurs under a certain condition. The first subscript is used to indicate the location of bites (I = indoors; O = outdoors; S = sleeping space). The second subscript is used to indicate whether the indicator is referring to someone who is protected by an ITN (p) during sleeping hours or unprotected throughout the night (u). The following equations are intended to reflect a twenty-four-hour period to account for mosquito biting activity and human behaviours over the course of a full day, although for practical purposes hours of full daylight in which *Anopheles* malaria vectors in most settings are inactive may be assumed to be negligible. An Excel^®^ spreadsheet template is provided to calculate these indicators from raw data using the examples presented in Figs. 1 and 2 (See Additional file 1).

1. Proportion of vector bites occurring indoors for an unprotected individual $\left( {\pi_{I,u} } \right)$ [52, 54–56]: This is an indicator of the maximum possible protection any indoor intervention could provide and is expressed as the number of bites received indoors over a 24-h period divided by the total number of bites received indoors and outdoors over the same 24 h period. It is calculated as the sum of the measured indoor vector biting rates (*B*_*I*_) for each one-hour time period (*t*) over a 24-h period weighted by the estimated proportion of humans indoors (*I*) at that time, divided by the total location weighted exposure (Eq. 1 denominator), i.e. itself plus the sum of the outdoor biting rates weighted by the proportion of humans outdoors (*O*, where *O *= 1–*I*) at each time over the same 24-hour period:

1 $$\pi_{I,u\,} = \,\frac{{\sum\nolimits_{t = 1}^{24} {B_{I,t} \,I_{t} } }}{{\sum\nolimits_{t = 1}^{24} {B_{I,t} \,I_{t} } \, + \,B_{O,t} \,O_{t} }}$$

It may sometimes be useful to instead express this summary indicator as its complement, the proportion of bites occurring outdoors for an unprotected individual (*π*_*O,u*_) [19]:

2 $$\pi_{O,u} \, = \,1 - \pi_{I,u}$$

2. Proportion of vector bites occurring while asleep for an unprotectedindividual $\left({\pi_{S,u}}\right)$ [16, 52–54, 56]: An indicator of the maximum possible protection any intervention targeting indoor sleeping spaces could provide. It is expressed as the number of vector bites received while asleep indoors divided by the total number of bites received indoors and outdoors during a 24-h period. It is calculated as the sum of the indoor vector biting rates (*B*_*I*_) for each 1-h time period (*t*) over a 24-h period weighted by the estimated proportion of humans sleeping (*S*) indoors at that time, by the sum of the indoor and outdoor biting rates respectively weighted by the proportions of humans indoors and outdoors at each time over the same 24-h period:

3 $$\pi_{S,u} \, = \,\frac{{\sum\nolimits_{t = 1}^{24} {B_{I,t} \,S_{t} } }}{{\sum\nolimits_{t = 1}^{24} {B_{I,t} \,I_{t} } \, + \,B_{O,t} \,O_{t} }}\,$$

One important limitation of this calculation is that it assumes all sleeping spaces are indoors, which may not be the case in all settings.

3. Proportion of all vector bites directly prevented by using an ITN $\left( {P_{S}^{*} } \right)$ [43, 48, 51, 55]: An indicator of *the* de facto protection provided against all indoor and outdoor bites by using an ITN, allowing for non-use while awake and active. It is calculated as the product of the proportion of exposure occurring indoors while asleep and the personal protection against bites provided by an ITN while in use (*ρ*). Data on personal protection provided by an ITN while in use can be obtained from standardized experimental hut trials, in which it is referred to as *feeding inhibition.* Feeding inhibition is defined as the percentage of mosquitoes that are prevented from taking a blood meal out of all mosquitoes that would otherwise do so inside an experimental hut [83]. Weighting these estimates of personal protection while in use (*ρ*) by the patterns of relevant human and mosquito behaviours, the proportion of all vector bites prevented by using an ITN may be calculated as follows:

4 $$P_{S}^{*} \, = \,\rho \pi_{S,u} = \,\frac{{\rho \sum\nolimits_{t = 1}^{24} {B_{I,t} \,S_{t} } }}{{\sum\nolimits_{t = 1}^{24} {B_{I,t} \,I_{t} } \, + \,B_{O,t} \,O_{t} }}$$

4. Proportion of remaining exposure occurring indoors for a protected user of an ITN $\left( {\pi_{I,p}}\right)$ [54–56]: An indicator of how much of the remaining exposure occurs indoors for an individual who uses an ITN and where supplemental tools should be targeted (i.e. indoors, outdoors, or both). It is calculated by adjusting the estimate of $\pi_{I,u}$ to allow for the indoor personal protection provided by using an ITN:

5 $$\pi_{I,p} \, = \,\frac{{\left( {\sum\nolimits_{t = 1}^{24} {B_{I,t} \,I_{t} } } \right)\, - \,\rho \,\left( {\sum\nolimits_{t = 1}^{24} {B_{I,t} S_{t} } } \right)}}{{\left( {\sum\nolimits_{t = 1}^{24} {B_{o,t} \,O_{t} \, + \,B_{I,t} I_{t} } } \right)\, - \rho \left( {\sum\nolimits_{t = 1}^{24} {B_{I,t} S_{t} } } \right)}}$$

It may sometimes be useful to instead express this summary indicator as its complement, the proportion of bites occurring outdoors for an ITN user $\pi_{o,p}$ :

6 $$\pi_{O,p} = 1 - \pi_{I,p}$$

5. Population-wide mean personal protection against biting exposure provided by observed level of ITN use (*C*) in the community $\left( {P_{S,C}^{*} } \right)$: While ITNs can feasibly be used during sleeping hours, not all members of a population can or do use an ITN. This is an indicator of the *population*-*wide mean level of personal protection provided by current levels of ITN use*. Calculated simply as the product of the proportion of human population using an ITN each night and the overall personal protection provided by an ITN, allowing for the attenuating effects of exposure occurring when the user is active outside the net.

7 $$P_{S,C}^{*} = \frac{{\rho \sum_{t = 1 }^{24} B_{I,t } C_{t} }}{{\sum_{t = 1 }^{24} B_{I,t } I_{t} + B_{O,t} O_{t} }} = \rho \pi_{S,p} C$$

### Critical data elements

Measuring human exposure to malaria vectors requires timed estimates of indoor and outdoor vector densities as outlined below and representative estimates of the human population indoors and outdoors, awake and asleep, and using personal prevention measures, over the full period of vector feeding activity. Given the potential variation in both vector and human behaviour, it is important to capture this data across seasons when possible. It is also helpful to know the approximate level of personal protection, in terms of bite prevention, provided by an ITN during the times when it is used. Such entomological estimates of personal protection are typically expressed as proportional blood feeding reduction, defined as the percentage of mosquitoes that are prevented from taking a blood meal out of all mosquitoes that would otherwise do so inside an experimental hut. Suitable estimates for commonly-used products may be obtained from published experimental hut studies, ideally from the nearest and most relevant settings with similar vector populations [29]. Human behavioural data should include representative estimates of the proportion of the human population indoors versus outdoors, sleeping versus awake, and under the protection of an ITN throughout the night. In other words, where people are, whether they *could* feasibly use an ITN, and whether they *are* using an ITN. When possible, such human behaviour data should ideally be collected in a disaggregated format that is linkable to de-identified individual human study participants. This format allows aggregation into estimates for the mean proportion of the human population indoors, sleeping, and under the protection of an ITN at each time of the night, but also allows for assessment of the epidemiological importance of behavioural differences between individuals. A summary of recommended data elements is included in Box 2.

Box 2: Summary of critical entomological and human-behavioural data elements for quantifying the distribution of human exposure to malaria vectors across times of the night and indoor versus outdoor locations Entomological data:

- Local and directly comparable measurements of indoor and outdoor biting rates for individual vector species, separately for each hour over the full period of feeding activity.
- Reference estimates for the personal protection provided by ITNs while actually being used, expressed in terms of proportional human blood feeding reduction.

Human behavioural data:

- Local estimates of the proportions of the human population who are indoors versus outdoors for each hour of the night.
- Local estimates of the proportions of human population who are asleep or trying to sleep versus awake and active, for each hour of the night.
- Estimate of proportion of human population using an ITN for each hour of the night.

### Considerations for entomological data collection

Indoor and outdoor biting rates can be assessed through human landing catches (HLC) [30] or an alternative method verified to reproduce representative exposure density distributions by capturing host-seeking mosquitoes. While HLC has been traditionally referred to by entomologists as the “gold standard” method, it should not be undertaken lightly as a default method and may not be permitted in some contexts for ethical reasons, such as where arboviral transmission may occur. Before any alternative method can be considered adequate for surveying human exposure patterns it must first be compared with HLC to determine equivalence indoors versus outdoors, over the course of the night, across settings, and across seasons (Box 3).

None of the validation steps described in Box 3 is particularly difficult and a range of under-utilized alternatives to HLC exist that could be suitable [30, 31]. Although no exposure-free method has yet been identified that completely satisfies all the requirements described in Box 3, only slight discrepancies are observed between HLC and some of the latest electric grid (EG) trap prototypes [32, 33]. However, some limitations and uncertainties remain regarding the performance and reliability of EGs [34] and they have the potential to be somewhat laborious and hazardous to use in practice. More practical and reliable exposure-free alternatives to HLC requiring no electrical power source would be desirable in terms of convenience, scalability and safety. New double net trap designs, including a miniaturized version developed and applied to survey human exposure patterns indoors and outdoors, are emerging that might address this need once evaluated against the criteria in Box 3 [35].

One of the most commonly used methods for surveying malaria vector densities, the Centers for Disease Control and Prevention (CDC) light trap was originally designed for indoor use, generally catches fewer mosquitoes outdoors, and can have variable outdoor sampling efficiency between studies, seasons, and mosquito species [30]. It is, therefore, not suitable for comparing indoor and outdoor biting densities and thus not recommended for measuring the entomological indices described in Box 2.

An important priority for future research in this area should be the specific adaptation of safe, convenient methods for measuring the entomological metrics described in Box 2 and evaluating those mosquito trapping methods in terms of the performance criteria detailed in Box 3. In the meantime, investigators relying on EGs, double net traps, or any other alternative, should include such assessments in their study designs as an essential internal quality assurance component of their studies.

Collections should be carried out for all hours of vector feeding activity. This is referred to simply as “night” for the purposes of malaria vectors, although this may include early evenings and mornings. Ensuring that the fringes of mosquito activity are captured, which may require 14 h of collection or more in some locations, will improve accuracy of the measures of human-vector interactions and correct interpretations of data [19]. Given the importance of even minor fractions of outdoor exposure that may occur before dusk and after dawn to sustaining residual malaria transmission [3, 12, 15, 36], surveying even low levels of biting activity during these early and late stages of mosquito activity cycles is critical.

It is also important to be able to disaggregate biting rate profiles for individual vector species within taxonomic groups and complexes, many of which differ in their time of biting, preference for indoor or outdoor biting, and efficiency as vectors. This is especially important where the individual vector species contribute differentially to overall malaria transmission and also have different biting behaviour profiles as may be observed between *Anopheles gambiae* sensu stricto, *Anopheles arabiensis* and *Anopheles funestus* [4, 37, 38]. It is therefore important that the results of morphological or molecular identifications of individual specimens can be linked to the field data detailing where, when, and how they were captured [39]. While this may sound obvious, it is not the case for most malaria vector behaviour data. Indeed, only three published estimates for any of the summary indicators described in Box 1 unambiguously relate to a single, clearly identified species, rather than mixture of species within a complex or group [13].

Mosquito densities are variable across nights and locations, and the distribution across indoor and outdoor environments often changes according to season and micro-climatic conditions [40–42]. Mosquitoes may also shift host-seeking behaviours as an adaptive response to insecticidal pressure from indoor interventions [43]. Therefore, ensuring sufficient replicates across locations and seasons is key to establishing long-term trends in behaviour patterns of local mosquitoes.

Indoor and outdoor mosquito collections are generally carried out in the peri-domestic setting, inside and directly outside of homes, which may not accurately reflect exposure to malaria vectors when people are away from home. Therefore, in some contexts, collections should be considered in additional locations of interest within the community where people may be at risk of exposure, for instance forest camps, as well as among migrant and mobile populations [44, 45].

In addition to indoor and outdoor mosquito collections, estimates of human vector interactions require an estimate of personal protection provided by ITNs while in use. Context-specific, local measurements of biting reduction achieved by ITNs, mosquito-proofed housing, or other relevant protection measures are neither necessary nor realistic to expect for every setting. For most commonly used ITN products, reference estimates are available from several experimental hut evaluations across tropical Africa and Asia, although these may vary between products, locations and species. The most geographically and ecologically relevant of these published values can be used in the calculations described in Box 1, albeit with the caveat that variability may arise from context-dependent differences in the condition of these products under conditions of use in the real world.

In addition to the critical data elements outlined in Box 2, when possible, the environmental characteristics within each house used for indoor human landing catches, particularly in terms of repellents, irritants and physical barriers that reduce mosquito densities in the house should also be captured. In addition to any behaviour-modifying active ingredients used for ITNs and IRS, structural features such as closed eaves, complete ceilings, and screened windows, and wall surface substrate need to be carefully considered when collecting and interpreting data [46]. Additional variables that may influence biting patterns include habitual cooking locations, house size, and type of livestock present indoors and outdoors. While these fine details may not be necessary for population-level or district-level recommendations envisaged here, they can help to identify important sub-groups within the population, which may require specific recommendations.

Box 3: Criteria for validating alternative methods to human landing catches for quantitative assessments of human patterns of exposure to mosquito bites A method should ideally meet all criteria. If there are variances, they must be measured and adjusted for to achieve the necessary equivalency.

- **Relative performance compared to HLC must be consistent indoors and outdoors**. Note however, that because the derived metrics of human exposure are expressed and used as proportions, the method does not need to have equivalent absolute efficiency. If an alternative method is more (or less) efficient indoors than outdoors when compared to HLC, the method will yield correspondingly biased estimates of *where* human exposure occurs. As an example, in particular setting it may be acceptable if an alternative method catches only 30% of the number of mosquitoes obtained by HLC with the same sampling effort in the same times and places, so long as that relative efficiency is 30% indoors and 30% outdoors. Lessons may be learned from early efforts to develop customized EGs for this purpose; initial prototypes had higher relative efficiency outdoors than indoors [84], so further prototypes were developed to address these shortcomings [32, 33] and some discrepancies remain [34].
- **Those indoor and outdoor relative capture rates must remain constant throughout the night**. In simple terms, if both relative capture efficiencies average 30% over the course of the night, this needs to reflect steady estimates of 30% throughout the night. If instead this reflects the mean of an unstable device with 40% relative sampling efficiency at the start of the evening that declines to 20% by the following morning, the method will yield correspondingly biased estimates of *when* human exposure occurs. This criterion is of particular relevance to battery-powered traps [30]. Important lessons may be learned from early efforts to evaluate commercially-available electrocuting grids for this purpose; this approach failed because relative capture efficiency faded as battery charge drained over the course of the night [84, 85].
- **The capture method should have consistently density-independent sampling efficiency relative to HLC**. It is essential that a method is proven to satisfy this criterion, so that it can be reliably applied across a range of locations and seasons without compromising reliability or utility for comparing results. For example, if a method has a sampling efficiency of 30% relative to HLC when vector densities are low, it should also be 30% at high vector densities rather than tail off to 20% (or increase to 40%) as capture bags fill with mosquitoes, batteries drain faster, or human operators become overwhelmed. While this has not yet proven an issue for purpose-built electrocuting grids [84], the same cannot be said for CDC light traps [86].

### Considerations for human behavioural data collection

Different methods have been used to estimate the proposed human-behavioural data elements. To date, no “gold standard” has been established for collecting this data. Examples of methods used to measure human behaviour identified in the literature include direct observation [16, 47–50], providing a digital watch to a household member and having him/her record information for each household member at specified times [51], and using survey questions [16, 20, 38, 43, 48, 52–56]. When selecting a methodological approach, it is important to consider its strengths, limitations, feasibility, as well as potential biases in the chosen method. It is also important to consider the scale at which it can realistically be applied (village, district, or national) and whether it can provide individual-level data that can be disaggregated and linked to other complementary data such as malaria infection status, socioeconomic status, education, and other potential risk factors.

Survey questions have to date been the most widely used option for collecting the proposed human behavioural data elements. In addition to the standard survey questions on ITN use described in the current Roll Back Malaria Household Survey Indicators for Malaria Control [57], information on location and sleeping patterns throughout the night can be collected, for example using a set of five additional questions asked for each household member (Box 4). Variations of the survey questions put forward in this paper have been used in previous studies [16, 48, 52, 54, 56, 58], including one that triangulated the results of survey questions with direct observation data [48]. When survey questions are used, the phraseology of the questions and locally appropriate translations are crucial to ensure a clear understanding by study participants and accurate responses. Even when implemented well, there are a number of limitations associated with surveys, including the potential for bias in responses [59]. Additionally, using a small set of questions, such as those presented in Box 4, requires making assumptions to estimate an individual’s hourly location throughout the night, rather than measuring directly for each hour or time interval. For an individual who reported using an ITN the night before, this approach assumes he or she was under the protection of an ITN consistently from the time they reported sleeping until the time they reported waking up. Therefore, this approach inherently will not capture micro-level behaviours such as getting in and out of bed or removing an ITN during the night, sleeping up against the net, or leaving it partially open during the night [60, 61].

Direct observation of human behaviour by mosquito collector, household member, or trained data collector can provide a high level of detail on human behaviours throughout the night, compared to survey questions. However, reactivity, described as a change in behaviour due to the presence of an observer, must be considered [62]. This phenomenon tends to decrease over time suggesting the potential value of multiple nights of observation or an acclimation period [63, 64]. Certain groups or activities may be inherently easier to observe than others, which can bias results. If observations are taking place in the peri-domestic setting only, it is important to consider how time spent away from home will be measured and recorded. This is particularly important when occupations such as fishing, forest work, or migratory farming are practiced, as these may result in significant exposure to mosquito bites far from people’s homes [44]. When possible, data collection should be considered in additional locations where people spend significant time overnight, such as farm plots [65–67]. Community entry is critical to the success of observations. Therefore, prior to conducting observations, community leaders and household members should be fully briefed on the objectives of the study and the reason for observation. Further, observations require adequate supervision to ensure data quality, and if data collectors are from outside of the study community, a clear security plan to maintain safety throughout the night should also be put in place. The decision on who will carry out observations may depend on a number of factors including available resources and security. It is important to consider how the identity of the observer (household member, community member, or trained data collector) might impact the quality of the data as well as the behaviour of household members being observed.

There are a variety of platforms for collecting the critical data elements (Box 2) and programmes can select the option that works best for them based on the considerations outlined here. Platforms for collecting critical human behavioural data elements include routine entomological monitoring, national surveys, and stand-alone research studies. Collecting human behaviour data within entomological surveillance sites provides an opportunity to collect vector and human behaviour data within the same population sample and to track changes across seasons and over time. Many countries now conduct routine entomological surveillance, which often includes monitoring indoor and outdoor biting rates. Collecting a small set of human-behavioural variables (Box 2) in conjunction with ongoing entomological collections could provide an opportunity to improve the decision-making value of data from these programmatic surveillance platforms; if both types of data can be disaggregated down to matching 1-hour periods or disaggregated at least to small enough time intervals to intuit the potential for indoor interventions to protect community members.

Including a standard set of relevant questions in large-scale surveys (Box 4), such as malaria indicator surveys, provides the opportunity to collect information across a broad demographically and geographically representative sample, allowing comparison of human behaviour across settings without substantively increasing time or resource inputs. When using such national-scale survey platforms, it may not be possible to ensure the person(s) answering the survey questions is knowledgeable about household members’ behaviour, which represents a limitation of this option. Further, it is difficult to capture seasonal trends in human behaviour due to the intermittent, cross-sectional design of most large-scale surveys and entomological and human behavioural data may not be available from the same time and place.

Stand-alone research studies may also include layered entomological and human behavioural data, and it can be valuable to collect epidemiological data in the same population sample. This data can, and when possible, should be collected during epidemiological evaluations of vector control tools [26].

There are inherent trade-offs between the level of detail that can be obtained and the scale at which a method can be implemented. When possible, use of multiple methods and method triangulation of the results from such different, complementary approaches should be considered to mitigate limitations of individual methods.

Box 4: Suggested survey questions that can be used as part of a stand-alone survey or included as part of a national survey These questions can be asked in addition to standard survey questions on ITN use described in the current Roll Back Malaria Household Survey Indicators for Malaria Control. Defined categorical options should be offered as answers. For time-related questions 1, 2, 4 and 5, options are hour-long time periods starting every hour e.g. 18:00 to 19:00, 19:00 to 20:00 etc.

1. During what time period did [name] go to sleep yesterday?
2. During what time period did [name] wake up today?
3. Did [name] sleep indoors or outdoors last night?
4. If [name] slept indoors, during what time period did [name] finally go indoors for the evening last night?
5. If [name] slept indoors, during what time period did [name] first go outdoors for the day this morning?

### Considerations for statistical analysis and sample size calculation

The variability and reliability of these estimates should be presented using the 95% confidence interval calculated from data sets of adequate sample size, containing multiple observations of human and vector behaviour through space and time. It is possible to estimate sample sizes of proportions by classifying human behaviour or mosquito behaviour into categories. When considering the toolbox of vector control interventions that are currently available, appropriate classifications for human behaviour may be categorised as (1) indoors asleep, (2) indoors awake or (3) outdoors. Similarly, after estimating average bedtime as a cut off, vector mosquitoes may be classified by the proportion of bites that occur (1) indoors before bed, (2) indoors after bed (3) outdoors. While this is a simplification, it is adequate to allow the calculation of minimum sample sizes to precisely detect a specified difference at either the individual (household), or village (cluster) level provided some information on the variability in human and vector behaviour is available a priori to account for differences between villages [68]. Other methods commonly used for estimating mosquito data are simulation based methods, which detect true effect sizes for specified densities of mosquitoes biting indoors or outdoors as well as the expected statistical uncertainties [69]. Regardless of the method, data used for the sample size calculation should be obtained locally.

## Discussion

Ongoing transmission of malaria is a direct result of the overlap between human and vector behaviours, and intervention access and use. It is essential to look at these pieces together for a more complete picture of malaria exposure. This information can inform selection of appropriate vector control tools for implementation in a particular scenario, guide prioritization of interventions in resource-constrained environments, and allows for monitoring of temporal changes in performance of interventions that may be influenced by human and/or vector behaviour. For example, given the Kenya example illustrated in Figs. 1 and 2, programmes might wish to focus on indoor-oriented interventions to further reduce the indoor nighttime exposure to *An. arabiensis*. With increasing resistance to pyrethroids reported among malaria vectors throughout sub-Saharan Africa, continued indoor biting despite high coverage of ITNs may indicate a need to distribute piperonyl butoxide (PBO) or next generation nets. Likewise, monitoring would provide insights into to whether the proportion of exposure to vector bites occurring outdoors is stable or changing over time, as even small increases in outdoor exposure have the potential to impact transmission dynamics [12]. In other contexts, exposure patterns may point to the need for outdoor interventions to protect people when outdoors and awake, or to the importance of strengthening behaviour change interventions to increase ITN use during sleeping hours.

Longitudinal data across seasons can highlight the impact of seasonal variation in vector and/or human behavioural data. For example, in some contexts, people may spend more time outside when the weather is hot, compared to when the weather is cool or rainy [49, 70]. Outdoor sleeping and large-scale socio-cultural events may also increase during this time and sleeping patterns may differ during planting and harvesting seasons [49, 70]. ITN use can also vary seasonally due to factors such as heat, mosquito density, and perceived malaria risk [71]. Disaggregation of these indicators by age and sex can help programmes to further target interventions by demographic group, where needed. For example, in some settings, adolescent and adult males spend more time outdoors at night, are more likely to engage in nighttime livelihood activities, and more likely to sleep outdoors [11, 49, 70, 72]. These socio-demographic factors should be considered in combination with biological factors such as transmission intensity, vector density, and EIR when interpreting the data and considering how additional interventions could be targeted.

The approach presented in this paper provides improved estimates of biting risk by accounting for the availability of human hosts indoors and outdoors as well as the protection provided by an ITN or other personal protection measures. Beyond biting risk, there are wide-ranging applications for this approach. The key parameter values estimated by considering human-vector interaction can be used as input values and/or to validate host-vector interaction-based models, with the potential to improve their prediction accuracy [9, 73–76]. Transmission modeling integrates metrics that describe mosquito and human interaction, net use and IRS coverage, to holistically determine the efficacy of vector control interventions and the corresponding probable epidemiological impact [75–78]. These models make assumptions about the exposure to infectious bites that remains after deploying ITNs or IRS to a community that are already based on data such as those outlined above. Therefore, improving the quality and quantity of these data can help refine transmission and statistical model predictions of public health impact [1, 79].

Previous work has highlighted the importance of heterogeneity in entomological inoculation rates for malaria transmission [80] as well as the subsets of humans and mosquitoes contributing most to transmission [81]. The changes suggested for the collection of routine data that are made here will not directly address how heterogeneity in exposure risk within and between individuals across nights might affect malaria burden or control efforts. When possible, the suggested practices for collection of routine data should be implemented in a manner that allows these indicators to be directly linked to epidemiological outcomes like malaria incidence and prevalence, so that the importance of such underlying heterogeneities in exposure risk and intervention suitability can be better understood.

Further, while the approach presented here is useful for measuring patterns of human exposure to malaria vectors, complementary qualitative data is needed to characterize relevant nighttime activities, sleeping patterns, and intervention use in greater depth, to better understand groups that may be at higher risk, as well as to identify barriers and facilitators to malaria prevention in different contexts [49, 70, 82]. This information can be used to guide selection and deployment of context-appropriate interventions.

## Conclusions

Collecting and integrating a minimum set of human behavioural data elements–the proportion of the human population indoors, asleep, and using an ITN throughout the night–with hourly indoor and outdoor mosquito biting rates can provide a more accurate measure of when and where people are at risk and how best to protect them. This information can help to guide National Malaria Control Programmes in deploying interventions targeting specific vector and human behaviours and inform target product profiles for new tools [26]. If collected and used consistently, the critical data elements and indicators presented in this article can contribute to an improved understanding of how malaria transmission persists in the context of current interventions and how exposure patterns may change as new vector control tools are introduced, as well as the potential impact and limitations of these tools. This article is intended to consolidate understanding around work on this topic to date and provide a consistent framework for building upon it. Additional work is needed to address remaining questions, including further development and validation of methods for entomological and human behavioural data collection and analysis.

## Supplementary information

**Additional file 1: Figure S1a.** Directly measured biting rate and human location. **Figure S1b** and **S2a** Behavior-adjusted biting rate for an unprotected individual. **Figure S2b** Behavior-adjusted biting rate for an ITN user. **Figure S2c** Population-wide mean exposure to vector bites.

## Data Availability

All data generated or analysed during this study are included in this published article and its supplementary information files.

## Abbreviations

CDC: Centers for Disease Control and Prevention
IRS: Indoor residual spraying
ITN: Insecticide-treated net

## References

[1] Bhatt S, Weiss D, Cameron E, Bisanzio D, Mappin B, Dalrymple U (2015). The effect of malaria control on *Plasmodium falciparum* in Africa between 2000 and 2015. Nature.

[2] Killeen GF, Kiware SS, Okumu FO, Sinka ME, Moyes CL, Massey NC (2017). Going beyond personal protection against mosquito bites to eliminate malaria transmission: population suppression of malaria vectors that exploit both human and animal blood. BMJ Global Health.

[3] Killeen GF (2014). Characterizing, controlling and eliminating residual malaria transmission. Malar J.

[4] Durnez L, Coosemans M. Residual transmission of malaria: an old issue for new approaches. In: *Anopheles* mosquitoes: new insights into malaria vectors. Manguin S., Ed. IntechOpen, 2013:671–704.

[5] Elliott R (1968). Studies on man-vector contact in some malarious areas in Colombia. Bull World Health Organ.

[6] Garrett-Jones C. A method for estimating the man-biting rate. Geneva, World Health Organization; 1964. (https://apps.who.int/iris/handle/10665/65193).

[7] Elliott R (1972). The influence of vector behavior on malaria transmission. Am J Trop Med Hyg.

[8] Killeen GF, Seyoum A, Gimnig JE, Stevenson JC, Drakeley CJ, Chitnis N (2014). Made-to-measure malaria vector control strategies: rational design based on insecticide properties and coverage of blood resources for mosquitoes. Malar J.

[9] Kiware SS, Chitnis N, Devine GJ, Moore SJ, Majambere S, Killeen GF (2012). Biologically meaningful coverage indicators for eliminating malaria transmission. Biol Lett.

[10] Lindblade KA (2013). Does a mosquito bite when no one is around to hear it?. Int J Epidemiol.

[11] Monroe A, Moore S, Koenker H, Lynch M, Ricotta E (2019). Measuring and characterizing night time human behaviour as it relates to residual malaria transmission in sub-Saharan Africa: a review of the published literature. Malar J.

[12] Sherrard-Smith E, Skarp JE, Beale AD, Fornadel C, Norris LC, Moore SJ (2019). Mosquito feeding behavior and how it influences residual malaria transmission across Africa. Proc Natl Acad Sci USA.

[13] Killeen GF, Chaki PP, Reed TE, Moyes CL, Govella NJ. Entomological surveillance as a cornerstone of malaria elimination: a critical appraisal. In ‘Towards Malaria Elimination—A Leap Forward’. Manguin S, Dev V, Eds. IntechOpen, 2018.

[14] Killeen GF (2018). A revival of epidemiological entomology in Senegal. Am J Trop Med Hyg.

[15] Sougoufara S, Thiaw O, Cailleau A, Diagne N, Harry M, Bouganali C (2018). The impact of periodic distribution campaigns of long-lasting insecticidal-treated bed nets on malaria vector dynamics and human exposure in Dielmo, Senegal. Am J Trop Med Hyg.

[16] Huho B, Briët O, Seyoum A, Sikaala C, Bayoh N, Gimnig J (2013). Consistently high estimates for the proportion of human exposure to malaria vector populations occurring indoors in rural Africa. Int J Epidemiol.

[17] Barreaux P, Barreaux AM, Sternberg ED, Suh E, Waite JL, Whitehead SA (2017). Priorities for broadening the malaria vector control tool kit. Trends Parasitol.

[18] Durnez L, Coosemans M. Residual transmission of malaria: an old issue for new approaches. 2013. In ‘*Anopheles* mosquitoes–New insights into malaria vectors’. Manguin S, Ed. IntechOpen. 2013:671-704.

[19] Killeen GF, Seyoum A, Sikaala C, Zomboko AS, Gimnig JE, Govella NJ (2013). Eliminating malaria vectors. Parasit Vectors.

[20] Bradley J, Lines J, Fuseini G, Schwabe C, Monti F, Slotman M (2015). Outdoor biting by *Anopheles* mosquitoes on Bioko Island does not currently impact on malaria control. Malar J.

[21] Magesa S, Wilkes T, Mnzava A, Njunwa K, Myamba J, Kivuyo M (1991). Trial of pyrethroid impregnated bednets in an area of Tanzania holoendemic for malaria Part 2. Effects on the malaria vector population. Acta Trop.

[22] Gatton ML, Chitnis N, Churcher T, Donnelly MJ, Ghani AC, Godfray HCJ (2013). The importance of mosquito behavioural adaptations to malaria control in Africa. Evolution.

[23] Loll DK, Berthe S, Faye SL, Wone I, Koenker H, Arnold B (2013). User-determined end of net life in Senegal: a qualitative assessment of decision-making related to the retirement of expired nets. Malar J.

[24] Takken W (2002). Do insecticide-treated bednets have an effect on malaria vectors?. Trop Med Int Health.

[25] Waite JL, Swain S, Lynch PA, Sharma S, Haque MA, Montgomery J (2017). Increasing the potential for malaria elimination by targeting zoophilic vectors. Sci Rep.

[26] Killeen GF, Marshall JM, Kiware SS, South AB, Tusting LS, Chaki PP (2017). Measuring, manipulating and exploiting behaviours of adult mosquitoes to optimise malaria vector control impact. BMJ Global Health.

[27] Gleave K, Lissenden N, Richardson M, Choi L, Ranson H (2018). Piperonyl butoxide (PBO) combined with pyrethroids in insecticide-treated nets to prevent malaria in Africa. Cochrane Database Syst Rev.

[28] Hemingway J, Ranson H, Magill A, Kolaczinski J, Fornadel C, Gimnig J (2016). Averting a malaria disaster: will insecticide resistance derail malaria control?. Lancet.

[29] WHO. Guidelines for laboratory and field-testing of long-lasting insecticidal nets. Geneva, World Health Organization, 2013.

[30] Silver JB, Service MW (2008). Mosquito ecology: field sampling methods.

[31] Clements AN (1992). The biology of mosquitoes: development, nutrition and reproduction.

[32] Meza FC, Kreppel KS, Maliti DF, Mlwale AT, Mirzai N, Killeen GF (2019). Mosquito electrocuting traps for directly measuring biting rates and host-preferences of *Anopheles arabiensis* and *Anopheles funestus* outdoors. Malar J.

[33] Govella NJ, Maliti DF, Mlwale AT, Masallu JP, Mirzai N, Johnson PC (2016). An improved mosquito electrocuting trap that safely reproduces epidemiologically relevant metrics of mosquito human-feeding behaviours as determined by human landing catch. Malar J.

[34] Sanou A, Guelbéogo WM, Nelli L, Toé KH, Zongo S, Ouédraogo P (2019). Evaluation of mosquito electrocuting traps as a safe alternative to the human landing catch for measuring human exposure to malaria vectors in Burkina Faso. Malar J.

[35] Limwagu AJ, Kaindoa EW, Ngowo HS, Hape E, Finda M, Mkandawile G (2019). Using a miniaturized double-net trap (DN-Mini) to assess relationships between indoor–outdoor biting preferences and physiological ages of two malaria vectors, *Anopheles arabiensis* and *Anopheles funestus*. Malar J.

[36] Govella NJ, Ferguson H (2012). Why use of interventions targeting outdoor biting mosquitoes will be necessary to achieve malaria elimination. Front Physiol.

[37] Lwetoijera DW, Harris C, Kiware SS, Dongus S, Devine GJ, McCall PJ (2014). Increasing role of *Anopheles funestus* and *Anopheles arabiensis* in malaria transmission in the Kilombero Valley, Tanzania. Malar J.

[38] Russell TL, Govella NJ, Azizi S, Drakeley CJ, Kachur SP, Killeen GF (2011). Increased proportions of outdoor feeding among residual malaria vector populations following increased use of insecticide-treated nets in rural Tanzania. Malar J.

[39] Kiware SS, Russell TL, Mtema ZJ, Chaki P, Lwetoijera D, Chanda J (2016). A generic schema and data collection forms applicable to diverse entomological studies of mosquitoes. Source Code Biol Med.

[40] Ngowo HS, Kaindoa EW, Matthiopoulos J, Ferguson HM, Okumu FO (2017). Variations in household microclimate affect outdoor-biting behaviour of malaria vectors. Wellcome Open Res.

[41] Magbity E, Lines J (2002). Spatial and temporal distribution of *Anopheles gambiae s.l*. (Diptera: Culicidae) in two Tanzanian villages: implication for designing mosquito sampling routines. Bull Entomol Res.

[42] Smith T, Charlwood J, Takken W, Tanner M, Spiegelhalter D (1995). Mapping the densities of malaria vectors within a single village. Acta Trop.

[43] Thomsen EK, Koimbu G, Pulford J, Jamea-Maiasa S, Ura Y, Keven JB (2016). Mosquito behavior change after distribution of bednets results in decreased protection against malaria exposure. J Infect Dis.

[44] Gryseels C, Durnez L, Gerrets R, Uk S, Suon S, Set S (2015). Re-imagining malaria: heterogeneity of human and mosquito behaviour in relation to residual malaria transmission in Cambodia. Malar J.

[45] Durnez L, Mao S, Denis L, Roelants P, Sochantha T, Coosemans M (2013). Outdoor malaria transmission in forested villages of Cambodia. Malar J.

[46] Sherrard-Smith E, Griffin JT, Winskill P, Corbel V, Pennetier C, Djénontin A (2018). Systematic review of indoor residual spray efficacy and effectiveness against *Plasmodium falciparum* in Africa. Nat Commun.

[47] Bugoro H, Cooper RD, Butafa C, Iro’ofa C, Mackenzie DO, Chen C-C (2011). Bionomics of the malaria vector *Anopheles farauti* in Temotu Province, Solomon Islands: issues for malaria elimination. Malar J.

[48] Geissbühler Y, Chaki P, Emidi B, Govella NJ, Shirima R, Mayagaya V (2007). Interdependence of domestic malaria prevention measures and mosquito-human interactions in urban Dar es Salaam, Tanzania. Malar J.

[49] Monroe A, Asamoah O, Lam Y, Koenker H, Psychas P, Lynch M (2015). Outdoor-sleeping and other night-time activities in northern Ghana: implications for residual transmission and malaria prevention. Malar J.

[50] Finda MF, Moshi IR, Monroe A, Limwagu AJ, Nyoni AP, Swai JK (2019). Linking human behaviours and malaria vector biting risk in south-eastern Tanzania. PLoS ONE.

[51] Cooke MK, Kahindi SC, Oriango RM, Owaga C, Ayoma E, Mabuka D (2015). A bite before bed’: exposure to malaria vectors outside the times of net use in the highlands of western Kenya. Malar J.

[52] Bayoh MN, Walker ED, Kosgei J, Ombok M, Olang GB, Githeko AK (2014). Persistently high estimates of late night, indoor exposure to malaria vectors despite high coverage of insecticide treated nets. Parasit Vectors.

[53] Kamau A, Mwangangi JM, Rono MK, Mogeni P, Omedo I, Midega J (2018). Variation in the effectiveness of insecticide treated nets against malaria and outdoor biting by vectors in Kilifi, Kenya. Wellcome Open Res.

[54] Killeen GF, Kihonda J, Lyimo E, Oketch FR, Kotas ME, Mathenge E (2006). Quantifying behavioural interactions between humans and mosquitoes: evaluating the protective efficacy of insecticidal nets against malaria transmission in rural Tanzania. BMC Infect Dis.

[55] Moiroux N, Damien GB, Egrot M, Djenontin A, Chandre F, Corbel V (2014). Human exposure to early morning *Anopheles funestus* biting behavior and personal protection provided by long-lasting insecticidal nets. PLoS ONE.

[56] Seyoum A, Sikaala CH, Chanda J, Chinula D, Ntamatungiro AJ, Hawela M (2012). Human exposure to anopheline mosquitoes occurs primarily indoors, even for users of insecticide-treated nets in Luangwa Valley, South-east Zambia. Parasit Vectors.

[57] Household survey indicators for malaria control. MEASURE Evaluation, MEASURE DHS, President’s Malaria Initiative, Roll Back Malaria Partnership, UNICEF, World Health Organization. 2013. https://www.measureevaluation.org/resources/publications/ms-13-78. Accessed 15 Mar 2019.

[58] Msellemu D, Namango HI, Mwakalinga VM, Ntamatungiro AJ, Mlacha Y, Mtema ZJ (2016). The epidemiology of residual *Plasmodium falciparum* malaria transmission and infection burden in an African city with high coverage of multiple vector control measures. Malar J.

[59] Van de Mortel TF (2008). Faking it: social desirability response bias in self-report research. Aus J Adv Nurs.

[60] Harvey SA, Lam Y, Martin NA, Olórtegui MP (2017). Multiple entries and exits and other complex human patterns of insecticide-treated net use: a possible contributor to residual malaria transmission?. Malar J.

[61] Msellemu D, Shemdoe A, Makungu C, Mlacha Y, Kannady K, Dongus S (2017). The underlying reasons for very high levels of bed net use, and higher malaria infection prevalence among bed net users than non-users in the Tanzanian city of Dar es Salaam: a qualitative study. Malar J.

[62] Bernard HR (2012). Social research methods: Qualitative and quantitative approaches.

[63] Gittelsohn J, Shankar AV, West KP, Ram RM, Gnywali T (1997). Estimating reactivity in direct observation studies of health behaviors. Human Organization.

[64] Harvey SA, Olórtegui MP, Leontsini E, Winch PJ (2009). They’ll change what they’re doing if they know that you’re watching: measuring reactivity in health behavior because of an observer’s presence—a case from the Peruvian Amazon. Field Methods.

[65] Nonaka D, Laimanivong S, Kobayashi J, Chindavonsa K, Kano S, Vanisaveth V (2010). Is staying overnight in a farming hut a risk factor for malaria infection in a setting with insecticide-treated bed nets in rural Laos?. Malar J.

[66] Swai JK, Finda MF, Madumla EP, Lingamba GF, Moshi IR, Rafiq MY (2016). Studies on mosquito biting risk among migratory rice farmers in rural south-eastern Tanzania and development of a portable mosquito-proof hut. Malar J.

[67] Edwards HM, Sriwichai P, Kirabittir K, Prachumsri J, Chavez IF, Hii J (2019). Transmission risk beyond the village: entomological and human factors contributing to residual malaria transmission in an area approaching malaria elimination on the Thailand-Myanmar border. Malar J.

[68] Hayes R, Bennett S (1999). Simple sample size calculation for cluster-randomized trials. Int J Epidemiol.

[69] Johnson PC, Barry SJ, Ferguson HM, Müller P (2015). Power analysis for generalized linear mixed models in ecology and evolution. Methods Ecol Evol.

[70] Monroe A, Mihayo K, Okumu F, Finda M, Moore S, Koenker H (2019). Human behaviour and residual malaria transmission in Zanzibar: findings from in-depth interviews and direct observation of community events. Malar J.

[71] Koenker H, Taylor C, Burgert-Brucker CR, Thwing J, Fish T, Kilian A (2019). Quantifying seasonal variation in insecticide-treated net use among those with access. Am J Trop Med Hyg.

[72] Ahorlu CS, Adongo P, Koenker H, Zigirumugabe S, Sika-Bright S, Koka E (2019). Understanding the gap between access and use: a qualitative study on barriers and facilitators to insecticide-treated net use in Ghana. Malar J.

[73] Chitnis N, Hyman JM, Cushing JM (2008). Determining important parameters in the spread of malaria through the sensitivity analysis of a mathematical model. Bull Math Biol.

[74] Churcher TS, Trape J-F, Cohuet A (2015). Human-to-mosquito transmission efficiency increases as malaria is controlled. Nat Commun.

[75] Griffin JT, Hollingsworth TD, Okell LC, Churcher TS, White M, Hinsley W (2010). Reducing *Plasmodium falciparum* malaria transmission in Africa: a model-based evaluation of intervention strategies. PLoS Med.

[76] Kiware SS, Chitnis N, Tatarsky A, Wu S, Castellanos HMS, Gosling R (2017). Attacking the mosquito on multiple fronts: insights from the Vector Control Optimization Model (VCOM) for malaria elimination. PLoS ONE.

[77] Eckhoff PA (2011). A malaria transmission-directed model of mosquito life cycle and ecology. Malar J.

[78] Griffin JT, Bhatt S, Sinka ME, Gething PW, Lynch M, Patouillard E (2016). Potential for reduction of burden and local elimination of malaria by reducing *Plasmodium falciparum* malaria transmission: a mathematical modelling study. Lancet Infect Dis.

[79] Winskill P, Walker PG, Griffin JT, Ghani AC (2017). Modelling the cost-effectiveness of introducing the RTS, S malaria vaccine relative to scaling up other malaria interventions in sub-Saharan Africa. BMJ Global Health.

[80] Smith DL, McKenzie FE, Snow RW, Hay SI (2007). Revisiting the basic reproductive number for malaria and its implications for malaria control. PLoS Biol.

[81] Gonçalves BP, Kapulu MC, Sawa P, Guelbéogo WM, Tiono AB, Grignard L (2017). Examining the human infectious reservoir for *Plasmodium falciparum* malaria in areas of differing transmission intensity. Nat Commun.

[82] Monroe A, Harvey SA, Lam Y, Muhangi D, Loll D, Kabali AT (2014). “People will say that I am proud”: a qualitative study of barriers to bed net use away from home in four Ugandan districts. Malar J.

[83] Okumu FO, Moore SJ (2011). Combining indoor residual spraying and insecticide-treated nets for malaria control in Africa: a review of possible outcomes and an outline of suggestions for the future. Malar J.

[84] Maliti DV, Govella NJ, Killeen GF, Mirzai N, Johnson PC, Kreppel K (2015). Development and evaluation of mosquito-electrocuting traps as alternatives to the human landing catch technique for sampling host-seeking malaria vectors. Malar J.

[85] Majambere S, Massue DJ, Mlacha Y, Govella NJ, Magesa SM, Killeen GF (2013). Advantages and limitations of commercially available electrocuting grids for studying mosquito behaviour. Parasit Vectors.

[86] Briët OJ, Huho BJ, Gimnig JE, Bayoh N, Seyoum A, Sikaala CH (2015). Applications and limitations of Centers for Disease Control and Prevention miniature light traps for measuring biting densities of African malaria vector populations: a pooled-analysis of 13 comparisons with human landing catches. Malar J.

