# Correction to: Methods and indicators for measuring patterns of human exposure to malaria vectors

**DOI:** 10.1186/s12936-020-03308-3

**Published:** 2020-07-13

**Authors:** April Monroe, Sarah Moore, Fredros Okumu, Samson Kiware, Neil F. Lobo, Hannah Koenker, Ellie Sherrard-Smith, John Gimnig, Gerry F. Killeen

**Affiliations:** 1 Johns Hopkins Center for Communication Programs, PMI VectorWorks Project, Baltimore, MD USA; 2 University of Basel, Basel, Switzerland; 3 Swiss Tropical and Public Health Institute, Basel, Switzerland; 4 Environmental Health and Ecological Sciences Department, Ifakara Health Institute, Ifakara, Tanzania; 5 School of Public Health, Faculty of Health Sciences, University of the Witwatersrand, Parktown, Republic of South Africa; 6 Institute of Biodiversity, Animal Health and Comparative Medicine, University of Glasgow, Glasgow, UK; 7 Eck Institute for Global Health, University of Notre Dame, Notre Dame, IN USA; 8 MRC Centre for Global Infectious Disease Analysis, Department of Infectious Disease Epidemiology, Imperial College London, Norfolk Place, London, W2 1PG UK; 9 Division of Parasitic Diseases, Centers for Disease Control and Prevention, Atlanta, GA USA; 10 Department of Vector Biology, Liverpool School of Tropical Medicine, Liverpool, UK; 11 School of Biological, Earth & Environmental Sciences and Environmental Research Institute, University College Cork, Cork, Republic of Ireland

## Correction to: Malar J (2020) 19:207 10.1186/s12936-020-03271-z

Following publication of the original article [1], the authors reported an error in the formatting of Boxes 1, 3 and 4, affecting either the HTML or the PDF versions of the article.

To explain the errors further:

In the PDF version of the article:

- For Box 1, Eq. 7 appeared outside of the box, in the main article body.

While in the HTML version:

- For Box 3, the text beginning with “None of the validation steps…” (from the section “Considerations for entomological data collection”) appeared erroneously in the box instead of the main article body.
- For Box 4, the text beginning with “Direct observation of human behaviour…” (from the section “Considerations for human behavioural data collection”) appeared erroneously in the box instead of the main article body.

The original article [1] has now been updated to correct this.

The publisher apologizes for any inconvenience caused.
