# Correction: Methods and indicators for measuring patterns of human exposure to malaria vectors

**DOI:** 10.1186/s12936-023-04676-2

**Published:** 2023-09-13

**Authors:** April Monroe, Sarah Moore, Fredros Okumu, Samson Kiware, Neil F. Lobo, Hannah Koenker, Ellie Sherrard-Smith, John Gimnig, Gerry F. Killeen

**Affiliations:** 1 Johns Hopkins Center for Communication Programs, PMI VectorWorks Project, Baltimore, MD USA; 2 University of Basel, Basel, Switzerland; 3 Swiss Tropical and Public Health Institute, Basel, Switzerland; 4 Environmental Health and Ecological Sciences Department, Ifakara Health Institute, Ifakara, Tanzania; 5 School of Public Health, Faculty of Health Sciences, University of the Witwatersrand, Parktown, Republic of South Africa; 6 Institute of Biodiversity, Animal Health and Comparative Medicine, University of Glasgow, Glasgow, UK; 7 Eck Institute for Global Health, University of Notre Dame, Notre Dame, IN USA; 8 MRC Centre for Global Infectious Disease Analysis, Department of Infectious Disease Epidemiology, Imperial College London, Norfolk Place, London, W2 1PG UK; 9 Division of Parasitic Diseases, Centers for Disease Control and Prevention, Atlanta, GA USA; 10 Department of Vector Biology, Liverpool School of Tropical Medicine, Liverpool, UK; 11 School of Biological, Earth & Environmental Sciences and Environmental Research Institute, University College Cork, Cork, Republic of Ireland

**Correction: Malaria J (2020) 19:207** **https://doi.org/10.1186/s12936-020-03271-z**

Following publication of the article [1], the authors flagged that there were some minor errors in the formulas of Additional file 1. These errors have since been corrected in the file. As a result of this correction, Fig. 2, which uses the illustrative data in Additional file 1, has also been corrected: in panel c of the figure, the value '60%' has been updated to '58%'. The authors would like to highlight that this corresponding update to the figure does not affect how their article should be interpreted; however, to ensure accurate calculations, it is important to ensure you use the corrected file if inputting your own data. The authors thank you for reading this erratum and apologize for any inconvenience caused.
